# Nursing terminology for the care of people with respiratory diseases and Covid-19

**DOI:** 10.1590/1980-220X-REEUSP-2023-0124en

**Published:** 2024-05-13

**Authors:** Jacira dos Santos Oliveira, Josilene de Melo Buriti Vasconcelos, Rafaella Felix Serafim Veras, Valkênia Alves Silva, Larrissa Mariana Bezerra França, Deborah Helena Batista Leite

**Affiliations:** 1 Universidade Federal da Paraíba, Centro de Ciências da Saúde, Departamento de Enfermagem Clínica, João Pessoa, PB, Brazil.; 2 Universidade Federal da Paraíba, Centro de Ciências da Saúde, Programa de Pós Graduação em Enfermagem, João Pessoa, PB, Brazil.

**Keywords:** Nursing, COVID-19, Respiratory Diseases, Standardized Nursing Terminology, Enfermería, COVID-19, Enfermedades Respiratorias, Terminología Normalizada de Enfermería, Enfermagem, Covid-19, Doenças Respiratórias, Terminologia Padronizada em Enfermagem

## Abstract

**Objectives:**

To build a specialized nursing terminology for the care of people with respiratory diseases and Covid-19 or who have respiratory diseases after Covid-19, based on ICNP^®^.

**Method:**

Methodological study developed in two stages: (1) identification of the relevant concepts for the health priority chosen from the literature; (2) cross-mapping of the identified concepts with the concepts contained in ICNP^®^ version 2019/2020.

**Results:**

9460 terms were extracted from the literature, of which 4065 terms were excluded because they were not related to the object of study and 5395 were submitted to the mapping technique, resulting in 290 constant terms in the ICNP^®^ and 5134 non-constant terms. The constant terms were classified into the following axes: 120 in the Focus axis, 13 in Judgment, 48 in Action, 23 in Location, 38 in Means, eight in Time and one in Client. In addition, 36 nursing diagnoses/outcomes and three nursing interventions were mapped.

**Conclusion:**

The terminology will support the quality of care provided by the nursing team and the manual and electronic recording of patient data.

## RESUMO

**Objetivos:**

Construir uma terminologia especializada de enfermagem para o cuidado à pessoa com doenças respiratórias e Covid-19 ou que apresentou doenças respiratórias após a Covid-19, fundamentada na CIPE^®^.

**Método:**

Estudo metodológico desenvolvido em duas etapas: (1) identificação dos conceitos relevantes para a prioridade de saúde escolhida a partir da literatura; (2) mapeamento cruzado dos conceitos identificados com os conceitos constantes na CIPE^®^ versão 2019/2020.

**Resultados:**

Foram extraídos 9460 termos a partir da literatura. Desse total, 4065 termos foram excluídos por não estarem relacionados ao objeto de estudo e 5395 foram submetidos à técnica de mapeamento, resultando em 290 termos constantes na CIPE^®^ e 5134 termos não constantes. Os termos constantes foram classificados nos eixos: 120 no eixo Foco, 13 no Julgamento, 48 no Ação, 23 no Localização, 38 no Meios, oito no Tempo e um no Cliente, além disso foram mapeados 36 diagnósticos/resultados de enfermagem e três intervenções de enfermagem.

**Conclusão:**

A terminologia subsidiará na qualidade da assistência da equipe de enfermagem e nos registros manual e eletrônico dos dados dos pacientes.

## RESUMEN

**Objetivos:**

Construir una terminología enfermera especializada para el cuidado de personas con enfermedades respiratorias y Covid-19 o que presentan enfermedades respiratorias después de Covid-19, basada en la ICNP^®^.

**Método:**

Estudio metodológico desarrollado en dos etapas: (1) identificación de los conceptos relevantes para la prioridad sanitaria elegida a partir de la literatura; (2) mapeo cruzado de los conceptos identificados con los conceptos contenidos en la CIPN^®^ versión 2019/2020.

**Resultados:**

9460 términos fueron extraídos de la literatura, de los cuales 4065 términos fueron excluidos por no estar relacionados con el objeto de estudio y 5395 fueron sometidos a la técnica de mapeo, resultando 290 términos constantes en la ICNP^®^ y 5134 términos no constantes. Los términos constantes se clasificaron en los siguientes ejes: 120 en el eje Enfoque, 13 en Juicio, 48 en Acción, 23 en Localización, 38 en Medios, ocho en Tiempo y uno en Cliente. Además, se mapearon 36 diagnósticos/resultados de enfermería y tres intervenciones de enfermería.

**Conclusión:**

La terminología va a subsidiar la calidad de los cuidados prestados por el equipo de enfermería y el registro manual y electrónico de los datos de los pacientes.

## INTRODUCTION

Coronavirus Disease 2019 (COVID-19) was identified in December 2019 in the city of Wuhan, Hubei province, China. It is a disease caused by Coronavirus 2 (SARS-CoV-2), a betacoronavirus leading to pneumonia^(1)^. Accordingly, people with respiratory diseases belonging to the risk group have become the target of greater attention from the country’s authorities and health professionals.

Covid-19 infection is akin to the clinical picture of a respiratory infection and the severity of symptoms ranges from a mild common cold to severe viral pneumonia, which can lead to a potentially fatal acute respiratory distress syndrome. Patients can be symptomatic or non-symptomatic, when they present symptoms they report fever, cough, dyspnea and more. Complications from Covid-19 can include multiple organ failure, septic shock and blood clots^(2)^.

Against this backdrop, nursing plays a key role in caring for people affected by Covid-19, and must guarantee them systematic, standardized and qualified care. To this end, they must comply with the legal precepts of the profession, applying the methodological instrument that is the Nursing Process^(3)^, guided by theories, models of care and standardized language systems that facilitate communication between their peers and other health professionals.

One of the standardized nursing terminologies is the International Classification of Nursing Practice – ICNP^®^, which is considered to be broad and complex, worldwide, and which includes primitive concepts to support the construction of statements of nursing diagnoses/outcomes and interventions, as well as pre-coordinated concepts^(4)^. The use of this classification is a cornerstone for the development of nursing, as it allows standards of care to be established that can be used anywhere in the world, as well as improving the quality of care and records and quantifying the activities carried out by the nursing team^(4)^.

A Brazilian study carried out with the aim of describing the use of ICNP^®^ in dissertations and theses from 2000 to 2018 identified 92 dissertations and 26 theses. This result shows the growth of academic productions on this classification^(5)^, which must continue to advance in order to support nursing practice.

In this same context, a study was identified in the literature involving the development of a bank of terms in the context of Covid-19 infections^(6)^ and another referring to a specialized nursing terminology for the care of people with Covid-19^(7)^, however, there are knowledge gaps related to the development of nursing terminologies aimed at patients with respiratory diseases and associated Covid-19. Covid-19 is a new disease for humanity, causing many early and unexpected deaths around the world, and for that reason, the scientific community continues to research in order to arrive at the specific treatment of the disease, since, at the moment, the greatest advances have been in prevention, notably with vaccines. Thus, the development of studies that make it possible to know the most prevalent concepts that characterize the relevant phenomena for Nursing in the care of adult and elderly people with respiratory diseases and Covid-19 or who have had respiratory diseases after Covid-19, are extremely relevant. Identifying these concepts will make it possible to compose a specific nursing terminology and will contribute to strengthening the electronic or manual health information system and will have an impact on the care provided to this clientele.

Considering the above, the aim of this study was to build a specialized nursing terminology for the care of adult and elderly people with respiratory diseases and Covid-19 or who presented respiratory diseases after Covid-19, based on ICNP^®^.

## METHOD

### Type of Study

This is a methodological study, following the model organized by Nóbrega et al.^(8)^ presented in two stages: (1) Identification of the concepts relevant to the health priority chosen from the literature and (2) Cross-mapping of the concepts identified with the concepts contained in CIPE^®^.

### Data Collection

In a first step, it was sought to identify the relevant concepts for caring for people with respiratory diseases and Covid-19 or who had respiratory diseases after Covid-19. A search was carried out according to the phases recommended by Ganong^(9)^, in the most relevant databases for Nursing: CINAHL, PubMed/MEDLINE, LILACS, EMBASE and SCOPUS. In addition, a search was made of the reference lists of the selected studies to see if there were any eligible references. It is important to note that this search was carried out by a group of independent reviewers.

The initial strategic simulation was: (MH “Respiratory Tract Diseases”) AND (MH “COVID-19/NU”) AND (year_cluster:[2020 TO 2021]). This simulation was modified depending on each database. Tools such as EndNote Web Basic (Clarivate Analytics^®^) and Rayyan were used to help remove duplicate articles, organize references and select articles.

Regarding the eligibility of the articles, the criteria used were based on the research question: What are the scientific evidences related to nursing care for people with respiratory diseases and Covid-19 or who have respiratory diseases after Covid-19, published in national and international journals? The inclusion criteria were original articles and studies/case reports; available in full; with a time-limit of 2020 to 2021 (the most critical period of the pandemic); Portuguese, English, Spanish and French languages; and the exclusion criteria: types of publication, such as literature reviews, conference abstracts, editorials, letters to the editor and book chapters; publications related to the topic with children and adolescents.

Data extraction from the primary studies was performed between September and December 2021 by selecting the articles and determining the corpus that allowed the main results to be extracted. An instrument validated by Toste and Galvão^(10)^ was used to extract data from the primary studies in order to compose the textual corpus, containing the title of the article, author(s), period, year of publication, objective(s), sample details, type of study, main results and conclusions, allowing for methodological rigor.

The primary studies were prepared for use in the PORONTO^(11)^ program, which is a free, open-source tool. This phase required attention and dedication in order to maintain efficiency in extracting the concepts. Twelve scientific articles were selected and went through a process of removing sections with low potential for relevant terms, such as authors, information about the authors, footnotes and references. The articles were then grouped into a single Word^®^ file, which was formatted and converted into a PDF file, forming the corpus of the study, called the “Literature Document in the area”. The extraction process began by sending the “Literature Document” file to the PORONTO tool, which automatically processed the file.

Among the terms extracted from the data processing result, simple terms and compound terms were selected, such as nouns, verbs, adverbs, verbal locutions and adverbial locutions, generating a list of terms organized in alphabetical order. The next step was to export the result into an Excel^®^ spreadsheet containing the list of terms from the literature. After this stage, Excel for Windows was used for normalization and standardization, with analysis and exclusion of some terms that were not necessary or important for the purpose of the study.

The spreadsheet resulting from data processing in PORONTO generated 9460 terms, which were evaluated by a group of three researchers in the study through manual screening and consensus. Of the terms evaluated, some were excluded because they were not related to the object of study, or because they were symbols or characters that did not represent terms, and the others were selected to be submitted to the cross-mapping technique (second step of the study).

### Data Analysis and Processing

The mapping according to ISO/TR12300:2016^(12)^ was of the human mapping type, in a one-way direction, starting from the terms extracted from the corpus to the terms of the Seven Axes Model of CIPE^®^ 2019/2020. This type of mapping allows the use of computer tools as support, so two spreadsheets were created in Excel for Windows, one containing the terms evaluated by the researchers and the other containing the terms from CIPE^®^ 2019/2020 (pre-coordinated concepts and terms from the CIPE^®^ 2019/2020 Seven Axes Model). These two spreadsheets were imported into the Access for Windows program to cross-check the data, resulting in terms that were present (constant terms) and those that were not present in CIPE^®^ 2019/2020 (non-constant terms).

The concepts were then standardized in terms of spelling and repetitions in order to ascertain their suitability, followed by a similarity analysis in which the concepts were compared with the Classification, and those that were similar (their meaning is identical) were replaced with the equivalent terms from CIPE^®^ 2019/2020. At the end of the second step, the nursing terminology for the care of people with respiratory diseases and Covid-19 was constructed.

The results were analyzed descriptively and presented in tables according to the characteristics of the data in absolute frequency. The results were interpreted against the backdrop of the relevant literature.

### Ethical Aspects

Regarding the issue of the Research Ethics Committee, under Resolution 510 of the National Health Council, of April 7, 2016, there was no need to apply to this study as it is a methodological research, using data in the public domain.

## RESULTS

Of the 12 articles selected from the literature, ten were in English, one in Portuguese and one in French. From these, 9460 terms were extracted and evaluated. Of these, 4065 were excluded because they were not related to the object of study and 5395 were submitted to the cross-mapping technique. Cross-mapping the 5,395 terms with ICNP^®^ 2019/2020 resulted in 290 constant terms (252 simple terms and 38 compound terms) in ICNP^®^ (Chart 1) and 5,134 non-constant terms that will not be presented in this study.

**Chart 1:** Simple terms and pre-coordinated concepts contained in CIPE^^®^^ 2019/2020 – João Pessoa, PB, Brazil, 2023.

Axis	Constant terms (N = 290)
**Diagnosis/Nursing Outcomes** (n = 36/12, 4%)*	Agitation (10007512), Anxiety (10007512), Apnea (10007512), Aspiration (10007512), Bradycardia (10007512), Health Seeking Behavior (10007512), Pain Control (10007512), Discomfort (10007512), Hopelessness (10007512), Disorientation (10007512), Diarrhea (10007512), Dyspnea (10007512), Pain (10013950), Fever (10007916), Hyperglycemia (10007512), Hyperthermia (10007512), Hypoglycemia (10007512), Infection (10007512), Inflammation (10007512), Tissue Integrity (10007512), Nausea (10007512), Fall (10007512), Rabies (10007512), Breathlessness (10007512), Risk of Aspiration (10007512), Risk of Bleeding (10007512), Risk of Infection (10007512), Risk of Injury (10007512), Risk of Loneliness (10007512), Risk of Suicide (10007512), Suffering (10007512), Suspicion (10007512), Tachycardia (10007512), Cough (10007512), Sadness (10007512), Vomiting (10020864).
**Nursing Intervention** (n = 3/1, 0%)	Oxygen Therapy (10007512), Occupational Therapy (10051282), Respiratory Therapy (10051586).
**Focus** (n = 120/41, 3%)	Self-Control (10017690), Self-Care (10017661), Self-Monitoring (10052146), Autonomy (10003054), Bradycardia (10007512), Chills (10018045), Shock (10018050), Septic Shock (10017898), Health-Seeking Behavior (10007512), Psychological Condition (10038430), Contamination (10025369), Pain Control (10005157), Healing (10008707), Discomfort (10023835), Hopelessness (10009105), Diabetes (10005876), Diarrhea (10005933), Dyspnea (10006461), Pain (10013950), Muscle Pain (10012316), Edema (10041951), Embolism (10051823), Family Coping (10034736), Sputum (10018717), Stress (10018888), Expectoration (10007362), Fever (10007916), Respiratory Rate (10016904), Bleeding (10008954), Hand Hygiene (10041190), Hyperglycemia (10027521), Hyperlipidemia (10041055), Hypertension (10009394), Hyperthermia (10009409), Hypoglycemia (10027513), Hypotension (10009534), Hypoxia (10009608), Infection (10010104), Cross Infection (10005404), Inflammation (10010127), Skin Integrity (10018241), Tissue Integrity (10003530), Death (10005560), Nausea (10012453), Obesity (10013457), Obstruction (10013555), Smell (10018327), Organism (10013783), Orientation (10013810), Taste (10019458), Role (10017321), Thought (10019663), Perception (10014270), Weight (10021034), Politics (10014726), Worry (10015466), Pressure (10015608), Blood Pressure (10003335), Procedure (10034409), Process (10015762), Fall (10007512), Anger (10002320), Recovery (10016507), Reflex (10016582), Regime (10016609), Diet (10046386), Regurgitation (10016632), Relationship (10016684), Resilience (10050418), Resistance (10006875), Breathlessness (10033334), Responsiveness (10017091), Result (10017186), Rhythm (10017210), Routine (10017384), Bleeding (10003303), Blood (10003319), Health (10008711), Dry (10006305), Secretion (10017635), Sedation (10040156), Service (10017908), Sign (10018130), Symptom (10019368), Socialization (10018391), Suffering (10019055), Loneliness (10011417), Sleep (10041399), Suction (10019001), Suicide (10019072), Sweat (10014449), Supply (10019119), Susceptibility(10019296),Suspicion(10019310), Tachycardia (10019415), Rate (10016390),Mortality Rate (10005573), Temperature (10019556), Body Temperature (10003507), Thermoregulation (10019644), Cough (10005249), Sadness (10017418),Gas Exchange (10008309), Ulcer (10020237), Urine (10020478), Value (10020599), Ventilation (10020704), Surveillance (10002144), Link (10003548), Vision (10018124), Victim (10042168), Vomit (10020864).
**Judgment** (n = 13/4, 4%)	Abnormal (10013269), Dependence (10026671), Partial (10014081), Small (10018315), Harmed (10012938), Prescribed (10015506), Presence (10046624), Progress (10015789), Real (10000420), Risk(10015007), Simple (10024061), Size (10018218), Total (10019876).
**Action** (n = 48/16, 5%)	Relieve (10002171), Analyze (10002298), Aspirate (10002641), Attend (10002911), Auscultate (10003012), Collect (10004574), Control (10005142), Disinfect (10006044), Wean (10020990), Document (10006173), Examine (10007256), Mobilize (10012120), Monitor (10012154), Observe (10013474), Obtain (10013572), Offer (10050313), Organize (10013806), Guide (10019502), Optimize (10013712), Listen (10011383), Participate (10014099), Allow (10014408), Weigh (10021023), Plan (10014648), Position (10014757), Prepare (10015478), Prescribe (10015523), Push (10015599), Prevent (10015620), Prioritize (10015736), Promote (10015801), Protect (10015864), Reinforce (10016650), Record (10016498), Regulate (10016613), Report (10016771), Remove (10016763), Respond (10017004), Supervise (10019093), Test (10019594), Transfer (10020030), Transfuse (10051670), Treat (10020133), Train (10020007), Exchange (10004162), Vaccinate (10020552), Vent (10020696), Turn (10020228).
**Location** (n = 23/7, 9%)	Artery (10002562), Bilateral (10027597), Heel (10008908), Capillary (10003860), Foot (10008155), Skin (10018239), Pelvis (10014236), Peripheral (10014386), Leg (10011298), Neck (10012476), Position (10014788), Body Position (10003433), Posterior (10014994), Prison (10015743), Lung (10015743), Wrist (10015743), Chin (10015743), Straight (10015743), Upper (10015743), Chest (10019692), Tracheotomy (10019951), University (10020302), Vein (10020665).
**Mean** (n = 38/13, 1%)	Alarm (10041491), Analgesic (10002279), Antibiotic (10002383), Antipyretic (10037253), Surgical Field (10019231), Cannula (10003856), Catheter (10004087), Urinary Catheter (10020373), Surgery (10019212), Device (10005869), Drain (10006207), Drug (10006314), Interprofessional Team (10039400), Insulin (10010400), Medication (10011866), Nutrient (10013398), Glasses (10008460), Oxygen Therapy (10007512), Plan (10014630), Protocol (10015926), Questionnaire (10016229), Meal (10011809), Bedding (10018499), Health Service (10008795), Solution (10018499), Suture (10019323), Technique (10019525), Respiratory Therapist (10051909), Therapy (10019628), Occupational Therapy (10000412), Respiratory Therapy (10037085), Tracheotomy (10019951), Pillow (10014607), Tube (10020216), Drainage Tube (10046109), Endotracheal Tube (10006868), Humidifier (10009228), Ventilator (10038430)
**Time** (n = 8/2, 7%)	Admission (10038430), Discharge (10038430), Chronic (10038430), Operation (10038430), Present (10038430), Week (10038430), Situation (10038430), Visit (10020817)
**Client** (n = 1/0, 3%)	Patient (10014132)

*Absolute and relative numbers.

Source: Research data.

Of the 290 terms in Chart 1, 29 were repeated both in the Focus Axis and in the pre-coordinated concepts of Nursing Diagnoses/Outcomes and one in Nursing Interventions, as shown in Chart 2.

**Chart 2:** Repeated terms in the Focus Axis and in the pre-coordinated concepts of Nursing Diagnoses/Outcomes and Nursing Interventions – João Pessoa, PB, Brazil, 2023.

Axis	Term
Focus and ND/NO*	Agitation, Anxiety, Apnea, Aspiration, Bradycardia, Health-seeking behavior, Pain control, Discomfort, Hopelessness, Diarrhea, Dyspnea, Pain, Fever, Hyperglycemia, Hyperthermia, Hypoglycemia, Infection, Inflammation, Tissue integrity, Nausea, Fall, Anger, Breathlessness, Suffering, Suspicion, Tachycardia, Cough, Sadness, Vomiting.
Mean and IC**	Oxygen Therapy

*ND/NO = Nursing Diagnoses/Nursing Outcomes.

**IC = Nursing Interventions.

Source: data from research.

Table 1 shows the 14 simple terms included in ICNP^®^ 2019/2020 with a repetition frequency greater than or equal to 30.

**Table 1:** Simple terms included in CIPE^®^ 2019/2020 with a repetition frequency greater than or equal to 30 – João Pessoa, PB, Brazil, 2023.

Term	F*	Term	F*
Patient	844	Signs	51
Position	156	Infection	50
Ventilation	100	Ulcer	49
Symptom	90	Observe	40
Risk	84	Ventilator	39
Oxygen therapy	62	Tube	38
Therapy	54	Chest	34

*F – absolute frequency.

Source: Data from research.

## DISCUSSION

The discussion is based on the construction of specialized nursing terminology for people with respiratory diseases and Covid-19.

The Nursing Diagnosis (ND) Single term “Infection” in ICNP^®^ 2019/2020 refers to the state of health of the person with Covid-19. The infection process causes a dry cough, upper airway congestion, fever, dyspnea, hypoxia, anosmia and ageusia, which can lead to the respiratory system being compromised to a severe state of the patient’s illness and is associated with the worst outcomes^(13)^.

“Oxygen therapy” is the term present in the axis: “Mean” and in the pre-combined concepts of the “Nursing Interventions” of ICNP^®^ 2019/2020. A similar result was found for the term “Mean” in a study on specialized nursing terminology for the care of people with Covid-19^(14)^. Whether as “Nursing Interventions” or as therapy, “Mean” is very important in acute respiratory failure (ARF) caused by Covid-19 in order to assist gas exchange in the supply and distribution of oxygen to cells, tissues and organs, reducing lung and tissue damage to the body^(15)^.

However, it is important to emphasize the importance of continuous nursing interventions that are consistent with the clinical condition of this disease, ensuring effective, safe and quality care. To this end, it is important for nursing professionals to keep up to date with a global health emergency in order to help cope with the disease^(16)^.

Among the terms on the “Focus” axis, two terms, “Ventilation” and “Symptoms” stand out, and the relationship between these terms is notorious. One study showed the need for mechanical ventilation as the primary outcome for patients affected by Covid-19 with symptoms of breathlessness, clinically confirmed by low saturation and blood gases^(17)^. Another study discussed the relationship between Covid-19 symptoms and the severity of clinical symptoms such as fever, fatigue and dyspnea, with complications associated with acute respiratory distress, which directly affect the patient’s ventilation^(18)^.

The term “ulcer” or “open wound or lesion” as defined in ICNP^®^ 2019/2020 may be one of the complications of using the prone position to improve oxygen saturation in patients with Covid-19 and that in this position, the person may be at a 22 times greater risk of developing a pressure injury^(19)^. Because of Covid-19, other risk factors for injury have been cited, such as vasoactive drugs and continuous sedation, the use of antibiotics, mechanical ventilation, enteral diet and/or zero diet, and length of stay.

This being said, it is possible to state that the constant terms found in the “Focus” axis are capable of guiding nurses in their decision-making behavior when caring for people affected by Covid-19, at different levels of complexity^(20)^.

Thus, the term “Risk” showed the highest frequency of appearance in the “Judgment” axis. This refers to the fact that the studies sought to identify clinical conditions or characteristics of the person that could predict the progression of the disease to a state of greater severity. Thus, there is a tendency to risk unfavorable prognoses in people with advanced age, comorbidities, respiratory diseases, smoking, cancer, diabetes and cardiovascular diseases^(21,22)^.

Accordingly, the term “Observe” in the “Action” axis was relevant for recognizing the state of severity. Observing signs and symptoms of severity can support referral to a more complex level of care or therapeutic decision-making by the health team. From this perspective, one study identified that people who progressed to a state of greater severity had a heterogeneous evolution and therefore required individualized care determined by observing their needs^(23)^.

In the “Location” axis, the constant term “Chest” was highlighted, which is related to the lung damage caused by Covid-19. Respiratory signs and symptoms are considered the main ones in the progression of the disease, both in mild and severe cases. Severe Acute Respiratory Syndrome is a complication associated with the need for intensive care and high mortality rates^(24)^. The nursing care described for performing the pronation maneuver and extracorporeal membrane oxygenation reflects the impact of the respiratory complications caused by the disease^(25)^.

Still on the “Location” axis, the term “Position” had one of the highest numbers of appearances in the selected articles. This term refers to the prone position of critically ill Covid-19 patients, which, if adopted early, has a significant effect on the hypoxemic level, resulting in improved oxygen saturation and a reduction in death cases^(26)^. It should be noted that the nursing team has played an important role in caring for patients in the prone position during the Covid-19 pandemic.

In the “Mean” axis, the constant term “Therapy” was the most relevant. It is extremely important that in the face of respiratory diseases, especially Covid-19, an effective therapy is defined that can reverse the signs and symptoms, promoting the recovery of the affected individual. To this end, it is necessary to implement institutional protocols that guide the way in which treatment is conducted, which should be based on proven evidence to guide the health team in making decisions, minimizing iatrogenesis and favoring the provision of standardized care in accordance with technical and scientific precepts^(27)^.

With regard to the constant terms “Ventilator and Tube”, also referring to the “Mean” Axis, it can be said that patients diagnosed with respiratory disease, and in particular with Covid-19, had a rapidly evolving clinical picture, especially among the groups listed as being at risk, in which one of the therapeutic tools used when the patient had persistent hypoxemic respiratory failure was endotracheal intubation and mechanical ventilatory support, with the aim of minimizing lung damage, such as atelectasis, preserving the breathing pattern and reversing the worsening of symptoms. This strategy was applied very frequently as part of the care protocol^(28)^.

The “Client” axis includes the constant term “Patient”, which refers to the way in which people who come to health institutions are conceptualized. Although the term refers to passivity, it should be emphasized that each individual has his or her own particularities, and nursing care should be dialogical, respectful and encourage the autonomy of the patient in different nuances, whether in promoting care or in making decisions according to their state of physical and mental integrity, establishing a relationship of trust between health professionals and client-patient^(29)^. A study carried out on specialized terminology for the clinical practice of people with Covid-19 showed a similar result for this Axis, presenting “Patient” as a constant term that must be seen in a holistic and respectful way, preserving their uniqueness and meeting their needs so that an effective therapeutic plan can be put together^(30)^.

In the context of the reports, it is understood that the constant terms are decisive for making decisions and implementing actions in a precise and specific way for the development of care practices, collaborating in the structuring of nursing diagnoses and their interventions based on clinical reasoning, promoting the operationalization of the Nursing Process.

The terms identified as non-constant reached a very significant number and were not presented in the study, constituting a limitation, but they should be analyzed in the future and taken into consideration in another research opportunity due to their importance for updating the ICNP^®^. These new terms identified through methodological research should be incorporated into the Classification.

The results of the study will imply the use of a standardized terminology in clinical nursing practice, providing nurses with autonomy in the care plan for adult and elderly people with respiratory diseases and Covid-19 or who have respiratory diseases after Covid-19, as well as contributing to the structuring of a CIPE^®^ terminological subset aimed at the aforementioned clientele.

## CONCLUSION

The specialized nursing terminology established in this study for the care of adult and elderly people with respiratory diseases and Covid-19 or who have respiratory diseases after Covid-19 can be considered an instrument in the nurse’s work process, supporting the recording of care in manual or electronic format in order to collaborate towards the standardization of a language among health professionals who care for a specific clientele with Covid-19.
